# Dentists’ Knowledge and Attitude Toward Tooth Autotransplantation in Saudi Arabia: A Cross-Sectional Survey

**DOI:** 10.3390/healthcare13131558

**Published:** 2025-06-30

**Authors:** Mohammad Assaggaf, Joweil Idrees, Maria Nassif, Shatha Bamashmous, Amal Jamjoom, Arwa A. Banjar, Arwa Badahdah, Ayman M. Abulhamael

**Affiliations:** 1 Department of Periodontology, Faculty of Dentistry, King Abdulaziz University, Jeddah 21589, Saudi Arabia; sbamashmous@kau.edu.sa (S.B.); agjamjoom@kau.edu.sa (A.J.); aazbangar1@kau.edu.sa (A.A.B.); aabadahdah@kau.edu.sa (A.B.); 2 Faculty of Dentistry, King Abdulaziz University, Jeddah 21589, Saudi Arabia; jjidrees@gmail.com (J.I.); mnassif98@gmail.com (M.N.); 3 Department of Endodontics, Faculty of Dentistry, King Abdulaziz University, Jeddah 21589, Saudi Arabia; amahmad4@kau.edu.sa

**Keywords:** attitude, autotransplantation, cross-sectional study, dentists, knowledge, questionnaire, survey

## Abstract

**Background/Objectives**: Extraction and replacement of hopeless teeth is a common practice in dentistry. Tooth autotransplantation (TA) offers several advantages as a viable and biological treatment option. However, its utilization in Saudi Arabia appears limited. Understanding dentists’ knowledge and attitudes toward TA is crucial for promoting its adoption in clinical practice. Therefore, the aim of this study is to assess the knowledge and attitudes of dentists in Saudi Arabia toward tooth autotransplantation. **Methods**: A cross-sectional study was conducted using a web-based questionnaire distributed to dentists across Saudi Arabia. The questionnaire included 19 questions assessing demographic characteristics, knowledge of TA, and attitudes toward its clinical application. Data were analyzed using SPSS v23 with chi-square and Cramér’s V tests to explore associations between variables (*p* < 0.05). **Results**: 253 dentists participated in this study. All participants had heard of TA, while only 26.5% reported moderate-to-high familiarity. Female dentists showed significantly greater interest in adopting TA than males (*p* = 0.038, Cramér’s V = 0.183). Specialists expressed higher familiarity than general dentists and underscored the importance of clinical guidelines, evidence-based outcomes, confidence in their capacity to engage in a TA team, the total number of required appointments, and malpractice concerns (*p* < 0.05) in their decision-making process. Nevertheless, general dentists demonstrated significantly more interest in implementing TA (*p* = 0.025, Cramér’s V = 0.192). Participants with more than 5 years of clinical dental experience were significantly more familiar with TA (*p* = 0.015, Cramér’s V = 0.204) and were more influenced by appointment numbers in decision-making (*p* = 0.012, Cramér’s V = 0.225). **Conclusions**: The study reveals limited familiarity but notable interest among dentists in Saudi Arabia toward TA. Addressing educational gaps by integrating TA training into dental curricula and offering clinical exposure opportunities to students, along with providing evidence-based clinical guidelines, and improving access to advanced imaging technologies, may enhance the adoption of TA as a viable treatment modality for tooth replacement.

## 1. Introduction

Premature loss of teeth due to dental caries or trauma is widely encountered among both adolescents and adults. The edentulous area is commonly restored using different types of tooth-supported or implant-supported prostheses. Alternatively, a viable method rarely used to replace non-restorable teeth is tooth autotransplantation (TA), a process in which the patient’s tooth is relocated to another socket [1]. Autotransplantation of third molars was first documented in 1954 by Apfel and Miller [2]. During tooth autotransplantation, it is essential to preserve the periodontal ligament (PDL) of the transplanted tooth to ensure the success of the procedure [3]. This can be accomplished by performing careful atraumatic extraction without jeopardizing the integrity of the periodontal ligaments and the underlying cementum, while temporarily storing the transplanted tooth in a suitable medium, such as Hank’s balanced solution.

Tooth autotransplantation provides several advantages over dental implants. A successful TA preserves alveolar bone density and maintains interdental papilla [4]. More significantly, this procedure can be used on growing-patient populations who are subjected to a higher incidence of dental trauma [5,6]. Following a successful transplantation, the transplanted tooth follows the normal eruption process; hence, it is particularly indicated for young patients. Pulp vitality, periodontal health, and proprioceptive function are maintained following a successful autotransplantation, as the PDL is formed around the transplanted tooth [7]. Tooth autotransplantation also enables orthodontic tooth movement, due to the preservation of the periodontal ligaments around the transplanted tooth, in contrast to ankylosed dental implants [8]. However, TA is a technique-sensitive procedure that often necessitates multidisciplinary involvement. For instance, it is recommended that an endodontist, periodontist, or oral surgeon, as well as a restorative dentist, be included in the TA team to plan and carry out the process from the transplantation day until the transplanted tooth is in function [9]. It is also susceptible to some relatively common complications. Pulpal necrosis usually occurs in mature transplanted teeth, so root canal therapy is often indicated to prevent inflammatory sequelae [10]. Furthermore, other complications, such as root resorption and ankylosis, though infrequent, remain potential risks that may compromise the clinical outcomes of this procedure [11]. A meta-analysis on the long-term prognosis of TA indicated an excellent survival rate of transplanted teeth, with ankylosis and root resorption occurring in less than 20% and 10% of cases, respectively [12]. This collectively underscores that careful patient selection and surgical technique are critical for TA success [13].

With recent advancements in oral and maxillofacial imaging technologies, preoperative cone beam computed tomography (CBCT) evaluation allows for comprehensive assessment of the donor tooth condition and recipient site suitability, as well as virtual planning of the procedure [14]. Digital intraoral scans can be combined with CBCT data to virtually simulate the transplant procedure and to fabricate the surgical guide, allowing precise visualization of the donor tooth’s fit and orientation in the recipient site [9]. Consequently, this increases the operator’s confidence and enhances the precision of the transplantation process. It also serves as a tool to facilitate the understanding of the procedure by the patient at the time of treatment planning. Overall, these advantages and innovations support the effective implementation of tooth autotransplantation, improve procedural predictability, and increase patient acceptance of this treatment option [15].

Tooth autotransplantation shows variable degrees of acceptance and adoption worldwide. For instance, it is more commonly practiced in parts of Europe and Japan. This could be attributed to well-constructed training protocols and the presence of clinicians more experienced in TA in these regions [16]. Meanwhile, TA is less frequently performed in Saudi Arabia. The inadequate incorporation of TA training within the curricula of dental programs in Saudi Arabia may contribute to its limited consideration as a treatment option among dentists in the country [17]. This educational gap was also shown among Syrian dentists [18] and maxillofacial residents in India [19].

In order to explore the dental community’s willingness and preparedness to adopt the TA technique as an option to replace a missing or non-restorable tooth, a cross-sectional survey assessing the knowledge and attitude of the dental community toward TA was conducted to help shed light on this issue. To our knowledge, no published studies have investigated the knowledge and attitudes of dentists regarding TA in Saudi Arabia. Only one recent cross-sectional study by Elbadawi et al. examined this topic, focusing specifically on dental interns in a single region of Saudi Arabia, and highlighting limited exposure to TA in undergraduate training [17]. Therefore, this study aims to address whether dentists in Saudi Arabia are knowledgeable about TA and have a positive attitude towards its clinical use. We hypothesized that, although most dentists in Saudi Arabia may have been introduced to the concept of TA, their familiarity and clinical exposure would be low due to minimal inclusion of TA in their dental training.

## 2. Materials and Methods

### 2.1. Study Design and Survey Instrument

This is a cross-sectional study utilizing a web-based survey, administered via Google Forms (Google LLC, Mountain View, CA, USA), as a data collection tool to assess the knowledge and attitude towards TA among dentists in Saudi Arabia. The survey used in the study is a modified version of that used by Dokova [20]. To establish the validity and reliability of the adapted instrument, multiple psychometric evaluations were conducted. Face and content validity were established through expert panel review during questionnaire development, and the modified questionnaire was pilot-tested. Construct validity was assessed using exploratory factor analysis, with a Kaiser–Meyer–Olkin (KMO) measure of 0.87 and a significant Bartlett’s test of sphericity (χ^2^ = 1495.593, df = 21, *p* < 0.001). Principal Component Analysis (PCA) with varimax rotation revealed a two-factor structure explaining approximately 81% of the total variance. Internal consistency of the Likert-scale items was high, with a Cronbach’s alpha of 0.93.

Ethical approval for this study was granted from the Research Ethical Committee at King Abdulaziz University, Jeddah, Saudi Arabia (040-01-23). Informed consent was obtained electronically from all participants prior to survey completion. The study ensured participant anonymity and data confidentiality in accordance with the ethical standards and the principles outlined in the Declaration of Helsinki. The survey included nineteen questions: six demographic questions (age, gender, years of experience, type of practice, and region), five questions assessing knowledge of tooth autotransplantation, and eight Likert-scale questions evaluating attitudes toward its clinical application. The survey questions are presented in Table A1. The sample size was calculated using the Raosoft online sample size calculator with a confidence level of 85%, a 5% margin of error, and an assumed response distribution of 50%. Although a 95% confidence level is widely accepted, an 85% confidence level was selected based on anticipated response rates to enable a feasible sample size for this study. Based on a total population of 27,181 dentists in Saudi Arabia, as reported by Alqahtani in 2022 [21], which was the most accessible estimate at the time of study planning, the required sample size was estimated at 205 participants.

### 2.2. Data Collection

A convenience sampling technique was utilized for data collection. The survey was distributed through different social media platforms to dentists across Saudi Arabia. Data were collected between February 2023 and January 2024. Only responses from dentists currently practicing in Saudi Arabia were included in the study. A total of 259 responses were received, of which 6 were excluded because the participants were either dental students or not practicing in Saudi Arabia.

### 2.3. Statistical Analysis

Statistical Package for the Social Sciences (SPSS) version 23 (IBM Corp., Armonk, NY, USA) was used to generate statistical analysis. Frequency distributions of demographics and dentists’ responses to survey questions were calculated. The chi-square and Cramér’s V tests were used to examine the differences in attitude and knowledge about TA between participants from different demographic backgrounds and between general dentists and specialists. A significance level of *p* value < 0.05 was used to indicate statistical significance.

## 3. Results

### 3.1. Study Participants

A total of 253 dentists met the inclusion criteria and participated in this study. The mean age of the participants was 35.2 ± 8.9 years. Table 1 presents the demographic characteristics of participating dentists, including gender, age, professional rank, postgraduate training, current practice type, region, and years of experience. The study sample consists of an almost equal distribution of males (49.8%) and females (50.2%), as well as general dentists (49.0%) and specialists (51.0%). Among specialists, the highest number of participants were restorative dentists (9.5%), pediatric dentists (9.1%), and prosthodontists (9.1%). Most participants were based in private practice (46.6%), with the majority of participating dentists practicing in the Western region (55.2%). With regards to years of experience, more than half of the participating dentists (53.4%) have been in practice for more than 5 years.

### 3.2. Dentists’ Knowledge and Attitudes Toward Tooth Autotransplantation

Table 2 presents the responses of participating dentists to survey questions assessing their knowledge and attitude regarding TA as a treatment option. The results show that all surveyed dentists had heard of TA, but only a small percentage reported being moderately familiar (19.8%) and very familiar (6.7%) with TA as a treatment option for missing teeth. While 54.5% of dentists reported being taught about TA during dental school, fewer received training during residency (29.6%) or in continuing education courses and workshops (22.9%).

Despite limited formal exposure, a considerable proportion of dentists expressed interest in incorporating TA into their clinical practice, with 24.1% reporting moderate interest and 12.6% demonstrating strong interest. Clinical outcomes were a key consideration in clinical decision-making: 73.6% of participants rated evidence-based outcomes as “very important” or “extremely important” in their decision to adopt TA. Similarly, the availability of clinical guidelines was considered very or extremely important by 77.5% of participants.

Other factors influencing dentists’ decisions to implement TA included confidence in their clinical capacity (rated “very important” or “extremely important” by 72%), proficiency in alternative tooth replacement procedures (68.4%), and patient expectations (66%). In contrast, the total number of required appointments was considered a less influential factor, with 45.4% rating it as “very important” or “extremely important.” However, malpractice liability remained a notable concern, with 70.3% of respondents identifying it as an important consideration in their decision-making.

### 3.3. Associations Between Demographic and Professional Characteristics and Dentists’ Knowledge and Attitudes Toward Tooth Autotransplantation

Table 3 presents the association between dentists’ knowledge about TA and their gender. The findings indicate that there were no statistically significant differences between males and females in terms of familiarity with autotransplantation, the sources of TA education received, rating the importance of various factors, and personal experience with the procedure. However, females were significantly more interested in implementing TA as a new treatment modality compared to males (*p* = 0.038, Cramér’s V = 0.183), indicating a weak association. Additionally, although male participants rated clinical outcomes based on evidence in the literature as more important in their decision-making compared to females, the difference was not statistically significant (*p* = 0.199).

When examining professional rank, several statistically significant differences were observed between general dentists and specialists, as presented in Table 4. Specialists demonstrated significantly greater familiarity with tooth autotransplantation (TA) (*p* = 0.001) and placed higher importance on multiple clinical decision-making factors. These included the availability of clinical guidelines (*p* = 0.008, Cramér’s V = 0.234), evidence-based clinical outcomes (*p* = 0.045, Cramér’s V = 0.196), confidence in their capacity to engage in a TA team (*p* = 0.043, Cramér’s V = 0.197), the total number of required appointments (*p* = 0.035, Cramér’s V = 0.203), and malpractice liability concerns (*p* = 0.043, Cramér’s V = 0.198). The corresponding Cramér’s V values indicate weak-to-moderate associations between professional rank and the perceived importance of these decision-making factors. Conversely, general dentists expressed significantly greater interest in implementing TA in their practice (*p* = 0.025, Cramér’s V = 0.192). Although specialists assigned more importance to their proficiency in other tooth replacement procedures compared to general dentists, this difference was not statistically significant (*p* = 0.097).

Table 5 presents the association between dentists’ knowledge and attitudes toward tooth autotransplantation (TA) and their years of clinical experience. Participants with more than 5 years of experience were significantly more familiar with TA compared to those with 0–5 years of experience (*p* = 0.015, Cramér’s V = 0.204). However, there was no significant association between years of experience and interest in implementing TA as a treatment modality (*p* = 0.266). Dentists with more than 5 years of experience were significantly more likely to consider the total number of required appointments as an important factor influencing their decision to adopt TA (*p* = 0.012, Cramér’s V = 0.225). No statistically significant differences were observed between the two experience groups for the remaining factors.

## 4. Discussion

Tooth autotransplantation (TA) is a viable treatment option for replacing a missing tooth by transferring the patient’s own sound tooth into the extraction site of the non-restorable tooth. Multiple case series published by Tsukiboshi et al. (2019) [22] and by Ong et al. (2021, 2023) [23,24] highlighted the importance of TA as a treatment option to replace missing teeth and showed successful long-term outcomes of TA. However, this treatment modality is not commonly considered or discussed by dentists when presenting options for replacing a hopeless tooth with their patients. Our study explored the knowledge and attitude toward TA among dentists in Saudi Arabia and revealed notable gaps in both familiarity and clinical training. The sample of dental professionals surveyed was diverse and representative of different aspects of the dental profession.

Our findings confirmed our initial hypothesis: while awareness of TA was virtually universal, the depth of knowledge and experience with TA among Saudi dentists was very limited. While all participants in this study were familiar with TA, the majority demonstrated limited familiarity, with only a small proportion expressing moderate or high familiarity. This aligns with previous studies reported by Elbadawi et al. (2023) and Nagori et al. (2016) [17,19], which identified insufficient exposure to TA during dental education as a key barrier. Structured training programs, such as those implemented in European countries and Japan, have been shown to improve clinicians’ confidence and proficiency in performing autotransplantation [16]. In contrast, the absence of dedicated educational modules on TA within Saudi dental curricula may explain the limited familiarity observed among participants in this study. These findings underscore the need to integrate TA into both undergraduate and postgraduate dental education programs in Saudi Arabia. In addition, consistent with Elbadawi et al. (2023), our results revealed a gender-related difference, with female participants expressing significantly more interest in implementing TA, possibly reflecting a stronger motivation to expand their clinical training in this area [17].

The underutilization of TA in Saudi Arabia reflects broader global trends observed in regions lacking established training protocols. Key barriers identified in this study include the absence of clinical guidelines, concerns regarding malpractice liability, and the perceived complexity of the procedure. Similar challenges have been reported in other studies [18,25,26], emphasizing the critical role of clear, evidence-based protocols in promoting clinical adoption of TA. Furthermore, participants emphasized the importance of patient-related factors, including expectations regarding cost, time, and long-term outcomes, in influencing their decision-making process. These findings underscore the need for targeted patient education and effective communication strategies to improve understanding and acceptance of TA as a practical treatment option. A study by Zhao et al. (2024) found limited knowledge but a generally positive attitude once informed of TA as an option when they surveyed patients with missing teeth in China [27].

Specialists showed significantly higher levels of familiarity with TA compared to general dentists, likely due to advanced training and exposure to complex clinical cases. However, general dentists expressed greater interest in adopting TA, suggesting a willingness to expand their clinical scope through continuing education. These findings are consistent with those of Kvint et al. (2010) [28], who highlighted the importance of hands-on workshops and mentorship programs in building clinician confidence and competence. Moreover, the limited familiarity observed may partly reflect that nearly half of the respondents had under five years of clinical experience. Encouraging collaboration between specialists and general dentists may further support knowledge transfer and promote the wider adoption of TA across various practice settings. Technological innovations, such as cone beam computed tomography (CBCT), digital intraoral scanners, and stereolithographic surgical guides, have significantly revolutionized the planning and execution of TA procedures. Those tools enable precise evaluation of both donor and recipient sites, reducing procedural risks and improving clinical outcomes [9,14,15]. However, despite these advancements, limited access to such technologies in certain practice settings may hinder the broader implementation of TA. Efforts to improve accessibility and provide targeted training on the use of advanced imaging tools could significantly enhance procedural predictability and increase clinician confidence in performing TA.

Overall, our study findings suggest that while TA may not be widely practiced or formally integrated into dental education in Saudi Arabia, it is an emerging treatment modality that dentists are interested in learning more about and potentially implementing in their practice. High adoption rates of TA have been reported in countries such as some European countries and Japan, likely due to well-established training protocols, greater clinician expertise, and the availability of comprehensive guidelines [16,19]. The development of similar frameworks in Saudi Arabia, including collaborations with international institutions, could help bridge the gap in knowledge and clinical practice. Our finding of limited familiarity of TA among dentists goes in line with the global trend. For instance, similar findings were reported among dentists in North Carolina [20]. Additionally, pediatric dentists in the city of Chennai in India were surveyed about their perceptions of autotransplantation, and showed inadequate training as the main reason for not adopting TA [29]. Also, a study exploring TA adoption among dentists in the city of Damascus in Syria revealed that limited knowledge and lack of experience were the main reasons for not adopting TA as a treatment option [18]

The findings of this study suggest several practical steps to enhance the adoption of TA in Saudi Arabia. For instance, incorporating TA into undergraduate and postgraduate curricula, with a focus on theoretical knowledge and clinical skills, could help improve familiarity with the procedure. Additionally, offering workshops and hands-on training programs for general dentists and specialists may support the development of confidence and clinical proficiency. Establishing evidence-based protocols is also advisable to standardize practice and decrease concerns regarding malpractice liability. Public awareness campaigns highlighting the benefits of TA, including its cost-effectiveness, biological advantages, and long-term outcomes, may further support its acceptance. Furthermore, improving the accessibility of CBCT and related technologies in dental practices, along with providing adequate training in their use, would be instrumental in supporting the successful implementation of TA. Based on recent observations [27], it is likely that many patients in Saudi Arabia are not yet aware of TA; however, they might prefer retaining their natural tooth over receiving an implant or denture if they were adequately informed. Dentists’ perceptions of patient acceptance may currently underestimate patients’ willingness to consider what is often viewed as an experimental procedure. 

This study has some limitations, including its cross-sectional design, dependence on a self-reported questionnaire and the use of a web-based survey distributed via social media, which may have introduced response and selection bias by excluding less digitally engaged dentists, and may have allowed participants to consult external sources during completion, potentially inflating self-reported familiarity with tooth autotransplantation. Additionally, the use of convenience sampling and a relatively limited sample size may restrict the generalizability of the findings to the wider dental population and may not fully reflect the diversity of dental practice and training across Saudi Arabia. While employing an 85% confidence level allowed for feasible sampling, it may have affected the precision and generalizability of the results. Furthermore, given the exploratory nature of this study, multiple bivariate chi-square tests were conducted without correction for multiple comparisons, increasing the risk of Type I error; therefore, marginally significant *p*-value should be interpreted with caution. Future studies may consider using a 95% confidence level to enhance the reliability of the findings. Moreover, future research may consider employing stratified random sampling using professional registries and recruiting larger, more representative samples, including multi-center or international cohorts, to determine whether the observed patterns of limited familiarity and interest in tooth autotransplantation are consistent across different settings. It could also explore the development and evaluation of targeted educational interventions, such as hands-on workshops and curricular integration, as potential strategies to enhance knowledge and encourage the clinical adoption of tooth autotransplantation.

## 5. Conclusions

This study highlights significant gaps in the knowledge and training of dentists in Saudi Arabia regarding tooth autotransplantation, emphasizing the need for targeted educational interventions and systemic changes to facilitate its adoption into clinical practice. By addressing these barriers, through improved curriculum content, hands-on training, clear clinical guidelines, and enhanced multidisciplinary support, the dental community can help utilize the biological and clinical benefits of TA. Implementing these measures could improve patient outcomes, especially for younger patients in whom implant treatment is relatively contraindicated, and expand treatment options available for managing tooth loss. We recommend that dental schools incorporate TA concepts and that professional authorities organize workshops and publish practice guidelines. Furthermore, increasing access to essential technologies, such as CBCT and digital planning tools, will be critical in enabling clinicians to successfully implement tooth autotransplantation as a viable modality in appropriate cases.

## Figures and Tables

**Table 1:** Demographic and Professional Characteristics of Participating Dentists (N = 253).

Variable		n (%)
**Gender**	Male	126 (49.8)
	Female	127 (50.2)
**Rank**	General dentist	124 (49.0)
	Specialist	129 (51.0)
**Postgraduate training**	AEGD	3 (1.2)
	Endodontics	14 (5.5)
	Oral maxillofacial surgery	13 (5.1)
	Orthodontics	10 (4.0)
	Pediatric dentistry	23 (9.1)
	Periodontics	10 (4.0)
	Prosthodontics	23 (9.1)
	Restorative	24 (9.5)
	Oral medicine	9 (3.6)
	No postgraduate training	124 (49.0)
**Current practice** **(Select all that apply)**	Educational sector	101 (39.9)
	Government	91 (36.0)
	Private practice	118 (46.6)
**Region**	Northern	36 (16.5)
	Central	44 (20.2)
	Southern	22 (10.1)
	Eastern	31 (14.2)
	Western	120 (55.2)
**Years of experience**	0–5	118 (46.6)
	>5	135 (53.4)

**Table 2:** Dentists’ Knowledge and Attitude Towards Autotransplantation (N = 253).

Question	Response	n (%)
Q. Have you heard of autotransplantation for replacement of missing teeth?	Yes	253 (100)
	No	0
Q. How familiar are you with tooth autotransplantation as a treatment option for patients with missing permanent teeth?	Not familiar	75 (29.6)
	Slightly familiar	111 (43.9)
	Moderately familiar	50 (19.8)
	Very familiar	17 (6.7)
Q. Were you taught concepts of autotransplantation in dental school?	Yes	138 (54.5)
	No	115 (45.5)
Q. Were you taught concepts of autotransplantation in graduate/residency training?	Yes	75 (29.6)
	No	178 (70.4)
Q. Were you taught concepts of autotransplantation in courses/workshops?	Yes	58 (22.9)
	No	195 (77.1)
Q. How interested are you in implementing autotransplantation as a new treatment modality in your practice?	Not interested	67 (26.5)
	Slightly interested	93 (36.8)
	Moderately interested	61 (24.1)
	Very interested	32 (12.6)
Q. Rate how important is availability of clinical guidelines in your decision to implement autotransplantation?	Not important	11 (4.3)
	Slightly important	21 (8.3)
	Neutral	25 (9.9)
	Very important	63 (24.9)
	Extremely important	133 (52.6)
Q. Rate how important are clinical outcomes based on evidence in the literature in your decision to implement autotransplantation?	Not important	10 (4.0)
	Slightly important	30 (11.9)
	Neutral	27 (10.7)
	Very important	72 (28.5)
	Extremely important	114 (45.1)
Q. Rate how important is confidence in your own capacity to engage in an autotransplantation team in your decision to implement autotransplantation?	Not important	8 (3.2)
	Slightly important	22 (8.7)
	Neutral	41 (16.2)
	Very important	92 (36.4)
	Extremely important	90 (35.6)
Q. Rate how important is your proficiency in other procedures and treatments to replace missing teeth in your decision to implement autotransplantation?	Not important	8 (3.2)
	Slightly important	26 (10.3)
	Neutral	46 (18.2)
	Very important	85 (33.6)
	Extremely important	88 (34.8)
Q. Rate how important is patient expectation of cost, time, long-term outcome in your decision to implement autotransplantation?	Not important	15 (5.9)
	Slightly important	29 (11.5)
	Neutral	42 (16.6)
	Very important	78 (30.8)
	Extremely important	89 (35.2)
Q. Rate how important is the total number of appointments in your decision to implement autotransplantation?	Not important	28 (11.1)
	Slightly important	39 (15.4)
	Neutral	71 (28.1)
	Very important	53 (20.9)
	Extremely important	62 (24.5)
Q. Rate how important is malpractice liability in your decision to implement autotransplantation?	Not important	14 (5.5)
	Slightly important	22 (8.7)
	Neutral	39 (15.4)
	Very important	79 (31.2)
	Extremely important	99 (39.1)

**Table 3:** Association Between Questionnaire Responses and Gender (N = 253).

Survey Questions on Knowledge and Attitudes		Gender			
		Male (%)	Female (%)	*p*-Value	Cramér’s V
How familiar are you with tooth autotransplantation as a treatment option for patients with missing permanent teeth?	Not familiar	(25.4)	(33.9)	0.494	0.097
	Slightly familiar	(47.6)	(40.2)		
	Moderately familiar	(19.8)	(19.7)		
	Very familiar	(7.1)	(6.3)		
Were you taught concepts of autotransplantation in dental school?	Yes	(54.8)	(54.3)	0.945	0.004
Were you taught concepts of autotransplantation in graduate/residency training?	Yes	(29.4)	(29.9)	0.923	0.006
Were you taught concepts of autotransplantation in courses/workshops?	Yes	(26.2)	(19.7)	0.218	0.077
How interested are you in implementing autotransplantation as a new treatment modality in your practice?	Not interested	(31.0)	(22.0)	0.038 *	0.183
	Slightly interested	(35.7)	(37.8)		
	Moderately interested	(26.2)	(22.0)		
	Very interested	(7.1)	(18.1)		
Rate how important is availability of clinical guidelines in your decision to implement autotransplantation?	Not important	(7.1)	(1.6)	0.127	0.168
	Slightly important	(7.1)	(9.4)		
	Neutral	(7.1)	(12.6)		
	Very important	(23.8)	(26.0)		
	Extremely important	(54.8)	(50.4)		
Rate how important are clinical outcomes based on evidence in the literature in your decision to implement autotransplantation?	Not important	(4.8)	(3.1)	0.199	0.154
	Slightly important	(11.9)	(11.8)		
	Neutral	(7.1)	(14.2)		
	Very important	(25.4)	(31.5)		
	Extremely important	(50.8)	(39.4)		
Rate how important is confidence in your own capacity to engage in an autotransplantation team in your decision to implement autotransplantation?	Not important	(4.0)	(2.4)	0.945	0.054
	Slightly important	(7.9)	(9.4)		
	Neutral	(16.7)	(15.7)		
	Very important	(36.5)	(36.2)		
	Extremely important	(34.9)	(36.2)		
Rate how important is your proficiency in other procedures and treatments to replace missing teeth in your decision to implement autotransplantation?	Not important	(4.0)	(2.4)	0.781	0.083
	Slightly important	(9.5)	(11.0)		
	Neutral	(16.7)	(19.7)		
	Very important	(36.5)	(30.7)		
	Extremely important	(33.3)	(36.2)		
Rate how important is patient expectation of cost, time, long-term outcome in your decision to implement autotransplantation?	Not important	(7.1)	(4.7)	0.846	0.074
	Slightly important	(10.3)	(12.6)		
	Neutral	(15.1)	(18.1)		
	Very important	(31.0)	(30.7)		
	Extremely important	(36.5)	(33.9)		
Rate how important is total number appointments in your decision to implement autotransplantation?	Not important	(12.7)	(9.4)	0.649	0.099
	Slightly important	(14.3)	(16.5)		
	Neutral	(24.6)	(31.5)		
	Very important	(23.0)	(18.9)		
	Extremely important	(25.4)	(23.6)		
Rate how important is malpractice liability in your decision to implement autotransplantation?	Not important	(7.9)	(3.1)	0.446	0.121
	Slightly important	(9.5)	(7.9)		
	Neutral	(13.5)	(17.3)		
	Very important	(29.4)	(33.1)		
	Extremely important	(39.7)	(38.6)		

Chi-square tests were used to assess differences between groups. *p*-value is significant at 0.05 level (*). Effect sizes were reported using Cramér’s V.

**Table 4:** Association Between Questionnaire Responses and Professional Rank (N = 253).

Survey Questions on Knowledge and Attitudes		Rank		*p*-Value	Cramér’s V
		General Dentists (%)	Specialists (%)		
How familiar are you with tooth autotransplantation as a treatment option for patients with missing permanent teeth?	Not familiar	(37.9)	(21.7)	0.001 *	0.265
	Slightly familiar	(46.8)	(41.1)		
	Moderately familiar	(12.1)	(27.1)		
	Very familiar	(3.2)	(10.1)		
Were you taught concepts of autotransplantation in dental school?	Yes	(59.7)	(49.6)	0.108	0.101
Were you taught concepts of autotransplantation in graduate/residency training?	Yes	(18.5)	(40.3)	<0.001 *	0.238
Were you taught concepts of autotransplantation in courses/workshops?	Yes	(23.4)	(22.5)	0.864	0.011
How interested are you in implementing autotransplantation as a new treatment modality in your practice?	Not interested	(28.2)	(24.8)	0.025 *	0.192
	Slightly interested	(32.3)	(41.1)		
	Moderately interested	(21.0)	(27.1)		
	Very interested	(18.5)	(7.0)		
Rate how important is availability of clinical guidelines in your decision to implement autotransplantation?	Not important	(4.8)	(3.9)	0.008 *	0.234
	Slightly important	(12.9)	(3.9)		
	Neutral	(12.1)	(7.8)		
	Very important	(28.2)	(21.7)		
	Extremely important	(41.9)	(62.8)		
Rate how important are clinical outcomes based on evidence in the literature in your decision to implement autotransplantation?	Not important	(4.8)	(3.1)	0.045 *	0.196
	Slightly important	(16.1)	(7.8)		
	Neutral	(13.7)	(7.8)		
	Very important	(28.2)	(28.8)		
	Extremely important	(37.1)	(52.7)		
Rate how important is confidence in your own capacity to engage in an autotransplantation team in your decision to implement autotransplantation?	Not important	(4.0)	(2.3)	0.043 *	0.197
	Slightly important	(13.7)	(3.9)		
	Neutral	(17.7)	(14.7)		
	Very important	(33.1)	(39.5)		
	Extremely important	(31.5)	(39.5)		
Rate how important is your proficiency in other procedures and treatments to replace missing teeth in your decision to implement autotransplantation?	Not important	(4.8)	(1.6)	0.097	0.176
	Slightly important	(12.9)	(7.8)		
	Neutral	(21.0)	(15.5)		
	Very important	(33.1)	(34.1)		
	Extremely important	(28.8)	(41.1)		
Rate how important is patient expectation of cost, time, long-term outcome in your decision to implement autotransplantation?	Not important	(7.3)	(4.7)	0.196	0.155
	Slightly important	(14.5)	(8.5)		
	Neutral	(19.4)	(14.0)		
	Very important	(29.0)	(32.6)		
	Extremely important	(29.8)	(40.3)		
Rate how important is total number appointments in your decision to implement autotransplantation?	Not important	(11.3)	(10.9)	0.035 *	0.203
	Slightly important	(21.0)	(10.1)		
	Neutral	(31.5)	(24.8)		
	Very important	(16.9)	(24.8)		
	Extremely important	(19.4)	(29.5)		
Rate how important is malpractice liability in your decision to implement autotransplantation?	Not important	(5.6)	(5.4)	0.043 *	0.198
	Slightly important	(12.1)	(5.4)		
	Neutral	(20.2)	(10.9)		
	Very important	(29.8)	(32.6)		
	Extremely important	(32.3)	(45.7)		

Chi-square tests were used to assess differences between groups. *p*-value is significant at 0.05 level (*). Effect sizes were reported using Cramér’s V.

**Table 5:** Association Between Questionnaire Responses and Years of Dental Experience (N = 253).

Survey Questions on Knowledge and Attitudes		Years of Experience			
		0–5 (%)	>5 (%)	*p*-Value	Cramér’s V
How familiar are you with tooth autotransplantation as a treatment option for patients with missing permanent teeth?	Not familiar	(36.4)	(23.7)	0.015 *	0.204
	Slightly familiar	(45.8)	(42.2)		
	Moderately familiar	(14.4)	(24.4)		
	Very familiar	(3.4)	(9.6)		
Were you taught concepts of autotransplantation in dental school?	Yes	(55.9)	(53.3)	0.679	0.026
Were you taught concepts of autotransplantation in graduate/residency training?	Yes	(24.6)	(34.1)	0.099	0.104
Were you taught concepts of autotransplantation in courses/workshops?	Yes	(25.4)	(20.7)	0.377	0.056
How interested are you in implementing autotransplantation as a new treatment modality in your practice?	Not interested	(23.7)	(28.9)	0.266	0.125
	Slightly interested	(35.6)	(37.8)		
	Moderately interested	(23.7)	(24.4)		
	Very interested	(16.9)	(8.9)		
Rate how important is availability of clinical guidelines in your decision to implement autotransplantation?	Not important	(5.1)	(3.7)	0.140	0.165
	Slightly important	(11.9)	(5.2)		
	Neutral	(12.7)	(7.4)		
	Very important	(22.9)	(26.7)		
	Extremely important	(47.5)	(57.0)		
Rate how important are clinical outcomes based on evidence in the literature in your decision to implement autotransplantation?	Not important	(5.9)	(2.2)	0.140	0.165
	Slightly important	(15.3)	(8.9)		
	Neutral	(12.7)	(8.9)		
	Very important	(26.3)	(30.4)		
	Extremely important	(39.8)	(49.6)		
Rate how important is confidence in your own capacity to engage in an autotransplantation team in your decision to implement autotransplantation?	Not important	(5.1)	(1.5)	0.067	0.186
	Slightly important	(12.7)	(5.2)		
	Neutral	(17.8)	(14.8)		
	Very important	(32.2)	(40.0)		
	Extremely important	(32.2)	(38.5)		
Rate how important is your proficiency in other procedures and treatments to replace missing teeth in your decision to implement autotransplantation?	Not important	(5.9)	(0.7)	0.131	0.168
	Slightly important	(11.9)	(9.9)		
	Neutral	(17.8)	(18.5)		
	Very important	(33.9)	(33.3)		
	Extremely important	(30.5)	(38.5)		
Rate how important is patient expectation of cost, time, long-term outcome in your decision to implement autotransplantation?	Not important	(8.5)	(3.7)	0.240	0.147
	Slightly important	(12.7)	(10.4)		
	Neutral	(19.5)	(14.1)		
	Very important	(28.8)	(32.6)		
	Extremely important	(30.5)	(39.3)		
Rate how important is total number appointments in your decision to implement autotransplantation?	Not important	(13.6)	(8.9)	0.012 *	0.225
	Slightly important	(20.3)	(11.1)		
	Neutral	(32.2)	(24.4)		
	Very important	(14.4)	(26.7)		
	Extremely important	(19.5)	(28.9)		
Rate how important is malpractice liability in your decision to implement autotransplantation?	Not important	(7.6)	(3.7)	0.078	0.182
	Slightly important	(11.0)	(6.7)		
	Neutral	(19.5)	(11.9)		
	Very important	(29.7)	(32.6)		
	Extremely important	(32.2)	(45.2)		

Chi-square tests were used to assess differences between groups. *p*-value is significant at 0.05 level (*). Effect sizes were reported using Cramér’s V.

## Data Availability

All raw supporting data will be available upon request.

## Abbreviations

The following abbreviations are used in this manuscript:

TA	Tooth Autotransplantation
PDL	Periodontal Ligaments
CBCT	Cone Beam Computed Tomography

## Appendix A

**Table A1:** Survey Questions.

Question	Responses
Q 1. What is your Gender?	Male
	Female
Q 2. What is your age?	
Q 3. How many total years have you been in practice?	
Q 4. Are you a:	General dentist
	Specialist
Q 5. In which province is your practice located?	
Q 6. Which of the following best describes your current practice type(s)? Select all that apply	Private practice
	Government
	Educational Sector
Q 7. Have you heard of autotransplantation for replacement of missing teeth?	Yes
	No
Q 8. How familiar are you with tooth autotransplantation as a treatment option for patients with missing permanent teeth?	Not familiar
	Slightly familiar
	Moderately familiar
	Very familiar
Q 9. Were you taught concepts of autotransplantation in dental school?	Yes
	No
Q 10. Were you taught concepts of autotransplantation in graduate/residency training?	Yes
	No
Q 11. Were you taught concepts of autotransplantation in courses/workshops?	Yes
	No
Q 12. How interested are you in implementing autotransplantation as a new treatment modality in your practice?	Not interested
	Slightly interested
	Moderately interested
	Very interested
Q 13. Rate how important is availability of clinical guidelines in your decision to implement autotransplantation?	Not important
	Slightly important
	Neutral
	Very important
	Extremely important
Q 14. Rate how important are clinical outcomes based on evidence in the literature in your decision to implement autotransplantation?	Not important
	Slightly important
	Neutral
	Very important
	Extremely important
Q 15. Rate how important is confidence in your own capacity to engage in an autotransplantation team in your decision to implement autotransplantation?	Not important
	Slightly important
	Neutral
	Very important
	Extremely important
Q 16. Rate how important is your proficiency in other procedures and treatments replace missing teeth in your decision to implement autotransplantation?	Not important
	Slightly important
	Neutral
	Very important
	Extremely important
Q 17. Rate how important is patient expectation of cost, time, long-term outcome in your decision to implement autotransplantation?	Not important
	Slightly important
	Neutral
	Very important
	Extremely important
Q 18. Rate how important is total number appointments in your decision to implement autotransplantation?	Not important
	Slightly important
	Neutral
	Very important
	Extremely important
Q 19. Rate how important is malpractice liability in your decision to implement autotransplantation?	Not important
	Slightly important
	Neutral
	Very important
	Extremely important
